# Maternal immunization with vaccines containing recombinant NetB toxin partially protects progeny chickens from necrotic enteritis

**DOI:** 10.1186/1297-9716-44-108

**Published:** 2013-11-13

**Authors:** Anthony L Keyburn, Ricardo W Portela, Mark E Ford, Trudi L Bannam, Xu X Yan, Julian I Rood, Robert J Moore

**Affiliations:** 1 CSIRO Biosecurity Flagship, Australian Animal Health Laboratory, Geelong 3220, Australia; 2 Australian Research Council Centre of Excellence in Structural and Functional Microbial Genomics, Department of Microbiology, Monash University, Clayton 3800, Australia; 3 Poultry Cooperative Research Centre, Armidale 2351, Australia; 4 Health Sciences Institute, Federal University of Bahia, Salvador, Brazil

## Abstract

Avian necrotic enteritis is a major economic and welfare issue throughout the global poultry industry and is caused by isolates of *Clostridium perfringens* that produce NetB toxin. Previously we have shown that birds directly vaccinated with inactivated *C. perfringens* type A culture supernatant (toxoid) combined with recombinant NetB (rNetB) protein were significantly protected from homologous and heterologous challenge. In the present study the protective effect of maternal immunization was examined. Broiler breeder hens were injected subcutaneously with genetically toxoided rNetB(S254L) alone, *C. perfringens* type A toxoid and toxoid combined with rNetB(S254L). Vaccination resulted in a strong serum immunoglobulin Y response to NetB in hens immunized with rNetB(S254L) formulations. Anti-NetB antibodies were transferred to the eggs and on into the hatched progeny. Subclinical necrotic enteritis was induced experimentally in the progeny and the occurrence of specific necrotic enteritis lesions evaluated. Birds derived from hens immunized with rNetB(S254L) combined with toxoid and challenged with a homologous strain (EHE-NE18) at either 14 or 21 days post-hatch had significantly lower levels of disease compared to birds from adjuvant only vaccinated hens. In addition, birds from hens immunized with rNetB(S254L) alone were significantly protected when challenged at 14 days post-hatch. These results demonstrate that maternal immunization with a NetB-enhanced toxoid vaccine is a promising method for the control of necrotic enteritis in young broiler chickens.

## Introduction

Necrotic enteritis in chickens has become a major welfare and economic issue for the global poultry industry in recent years [1]. Necrotic enteritis is a complex enteric disease and the causative agent is the Gram-positive, spore-forming bacterium *Clostridium perfringens*. The primary method of disease control has been to use ionophore anticoccidials or in-feed antibiotics [2]. However, there is global concern that these practices increase the level of bacterial antibiotic resistance [3]. Therefore, it has become increasingly important to develop new interventions for this disease, including the development of vaccines.

Clostridial vaccines in other livestock animals, including cattle, sheep and pigs, have been available for many decades [4], but an effective vaccine for necrotic enteritis in chickens remains limited with only one vaccine, Netvax®, currently commercially available. This vaccine is based on an alpha-toxin toxoid prepared from a *C. perfringens* type A strain isolated from a cow (CN 1491 alternately called ATCC 13124) [5]. Other experimental vaccines based on alpha-toxin have been evaluated with variable protective success [6]–[8] and other partially protective *C. perfringens* antigens have been identified [9]. Recently, several studies have demonstrated that the major toxin produced by pathogenic isolates of avian necrotic enteritis-derived *C. perfringens*, NetB, is an effective vaccine antigen [10]–[12]. These studies all demonstrated some protective efficacy when NetB was used as a single subunit vaccine, but in addition, it has been shown that rNetB in combination with other cellular and/or secreted antigens gave far greater protection against both homologous and heterologous challenge than any of the components used separately [12]. While these studies demonstrated that NetB based vaccines are effective in protecting birds from disease the delivery of the vaccines by direct vaccination of broiler chicks, is not a practical vaccination regime for the poultry industry. Attempts to vaccinate chickens at one day of age were not successful [13]. Maternal vaccination, by which broiler breeder hens are vaccinated to induce protection in the progeny, is a more efficient method of mass vaccinating large numbers of young birds.

For maternal vaccination to be successful antibodies passed from a vaccinated hen into the egg and the hatched chick must be capable of protecting the chicks from disease. The protective immune response mechanisms acting in parenteral vaccinated birds are not well understood. There are no clear studies that directly address the issue of whether passively transferred immunity can confer protection from disease. Two previously reported studies used *C. perfringens* toxoid vaccines in maternal vaccination trials and found some protection from necrotic enteritis. In one study [14] no details are provided regarding the origin of the strains used to produce the toxoids and in the other study [5] no details are given regarding the source of the toxoid, although a search of product registration documents reveals the strain to be derived from a cow rather than a chicken and not likely to have specific proteins involved in disease, such as NetB and host specific adhesions. The authors of each study suggested that antibodies against alpha-toxin were probably a significant protective factor. We hypothesized that if vaccines based on alpha-toxin, which is non-essential for virulence [15], could provide some level of protection from disease when maternally-delivered then maternal vaccines based on the essential virulence factor, NetB, should deliver enhanced efficacy.

Previously recombinant NetB was chemically toxoided for use in vaccines [12]. By using a mutated version of NetB with reduced toxic activity the need for chemical toxoiding can be avoided. Other workers [11] demonstrated that a genetically toxoided version of NetB(W262A) had similar efficacy in vaccination as wild-type recombinant NetB. A structural and mutational analysis of NetB has identified other amino acids that are important for toxic activity [16]. The substitution of serine residue 254 with leucine resulted in defective homo-oligomerization in solution and the mutant protein was no longer haemolytic against chicken and duck red blood cells.

The objective of this present study was to evaluate maternal immunization of vaccines containing NetB for the protection of broiler chickens from necrotic enteritis. We also determined serum IgY antibody responses to NetB in broiler breeder hens after immunization with genetically toxoided rNetB(S254L) alone or in combination with the other *C. perfringens* proteins present in culture supernatant (toxoid), and the transfer of NetB-specific antibody into progeny. Finally, the progeny were challenged in a disease induction model to evaluate protective immunity against necrotic enteritis lesion formation.

## Materials and methods

### Strain and chemicals

*C. perfringens* strain EHE-NE18 [15] was the challenge strain in the in vivo necrotic enteritis disease induction models and was used to prepare the toxoid for vaccine formulations.

### Animals and housing conditions

Twenty-week-old Ross 308 broiler breeder birds were supplied by Aviagen Australia and managed following the standard guidelines provided by the hatchery. The birds were housed in groups of 12 hens with 2 roosters per group. Immediately prior to the first vaccination blood samples were taken (wing brachial vein) and serum tested for anti-NetB specific IgY to determine whether there had been pre-exposure to this antigen. The hens were vaccinated subcutaneously at 22, 24 and 26 weeks of age. Blood and eggs were collected at 30 weeks (four weeks after the third vaccination) and serum and yolk anti-NetB specific IgY levels measured. Blood was also collected at 40 weeks for anti-NetB IgY measurement.

Fertilized eggs for Challenge Trial 1 were collected throughout week 31 and for Challenge Trial 2 throughout week 41. Eggs were stored at 17 °C throughout the collection period and then transferred to an incubator (Wesfan 924, WA, Australia) and then into a hatcher (Bellsouth, VIC, Australia) at day 18 till hatch.

### Vaccination

The toxoid was prepared as previously described [12]. Recombinant NetB(S254L) (rNetB(S254L)) was expressed and purified as previously described [16]. Fifty μg of rNetB(S254L) per dose per bird was used for NetB subunit and NetB supplemented toxoid vaccines. The vaccines were prepared in CSIRO triple adjuvant (60% (v/v) Montanide, 40% (v/v) antigen combined with Quil A, 3 mg/mL, and DEAE-dextran, 30 mg/mL in PBS) to a total volume of 500 μL per dose.

To assess the efficacy of the vaccines in the progeny from immunized hens at two susceptible time points (days 14 to 21), two challenge trials were carried out. In trial 1, fertile eggs were collected five weeks after the third vaccination (31 weeks old hens) and the progeny were challenged at days 21 and 22 followed by necropsy at day 23. In trial 2, fertile eggs were collected 15 weeks after the third vaccination (41 weeks old hens) and birds were challenged one week earlier than in trial 1 (challenged at day 14 and 15 followed by necropsy at day 16). In both trials there were 25 birds per group.

### Necrotic enteritis disease induction model

Necrotic enteritis was induced using the *C. perfringens* in-feed infection model as previously described [12]. Twenty-five chicks for each vaccine group were housed in adjacent, but separate, pens in an animal isolation facility. To assess the disease status of each chicken the birds were euthanized with inhaled carbon dioxide and small intestines (duodenum to ileum) were examined for gross necrotic lesions. Intestinal lesions in the small intestine were scored as before [15]: 0 = no gross lesions; 1 = thin or friable walls; 2 = focal necrosis or ulceration (1–5 foci); 3 = focal necrosis or ulceration (6–15 foci); 4 = focal necrosis or ulceration (16 or more foci); 5 = patches of necrosis 2–3 cm long; 6 = diffuse necrosis typical of field cases. The lesion data were analysed using Kruskal-Wallis one-way ANOVA to determine the likelihood that the vaccinated groups had different levels of disease compared to the adjuvant control group. A blood sample was taken from all birds immediately before challenge and serum was analysed by enzyme-linked immunosorbent assay (ELISA) to measure anti-NetB IgY antibody levels. All animal experiments were assessed, approved and monitored by the Australian Animal Health Laboratory’s Animal Ethics Committee.

### Immunoassay detection of antibodies

NetB-specific antibody levels in serum were determined by the end-point dilution method using an ELISA as described previously [12]. IgY antibodies were extracted from egg yolk as previously described [17], with some modifications. Briefly, the yolk was separated from the albumin and PBS was added at 1:1 ratio. The yolk was then vortexed till it reached a uniformly dispersed solution and was then homogenised for 30 min on a rotating wheel. The solution was centrifuged at 3000 *g* for 25 min and supernatant containing the IgY collected. The samples were diluted 1:1600 in 1% BSA in PBS and 0.1 mL per well was used in the anti-NetB IgY ELISA.

## Results

### Antibody transfer from vaccinated hens to progeny

**Figure 1:**
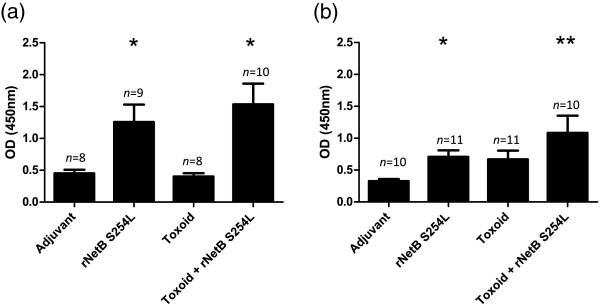
**Serum IgY antibody levels against *C. perfringens *NetB in hens after sub-cutaneous immunization with vaccines.** The levels of anti-NetB specific IgY antibodies were measured by ELISA and expressed as the average optical density at 450 nm of the standard dilution used. The error bars represent SEM. **(a)** Serum anti-NetB IgY levels of vaccinated hens at 30 weeks of age (4 weeks post final vaccination). **(b)** Serum anti-NetB IgY levels of vaccinated hens at 40 weeks of age (14 weeks post-final vaccination). *p < 0.05, **p < 0.01.

In broiler breeder hens, prior to the first vaccination, the mean anti-NetB IgY antibodies were at low, background levels in all birds (data not shown). Following three vaccinations the mean anti-NetB specific IgY antibody levels significantly increased in birds vaccinated with rNetB(S254L) (*p* < 0.05 at both 30 and 40 weeks; Figure 1a) and the toxoid supplemented with rNetB(S254L) (*p* < 0.05 and *p* < 0.01 at 30 and 40 weeks respectively; Figure 1b). Mean anti-NetB specific IgY antibodies of hens in the control (adjuvant only) and toxoid alone groups remained low throughout the study.

**Figure 2:**
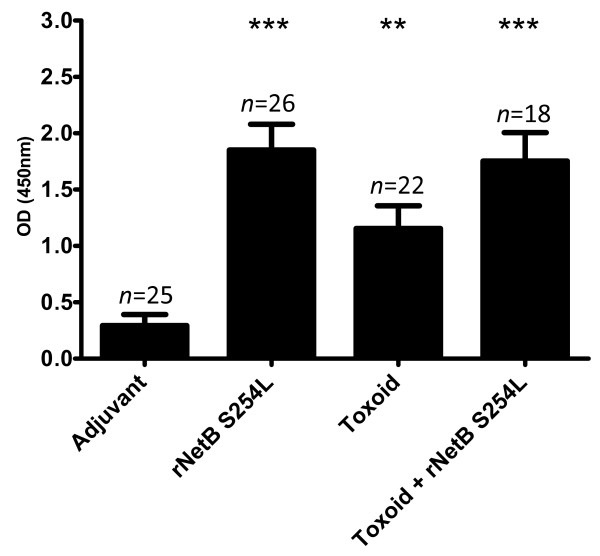
**Egg yolk anti-NetB IgY levels.** Fertilized eggs were collected from immunized hens at 30 weeks and specific antibodies derived from the egg yolk measured by ELISA. The error bars represent SEM. **p < 0.01, ***p < 0.001.

Fertilized eggs were collected at 30 weeks and tested for the transfer of anti-NetB specific IgY antibodies into the eggs. Higher anti-NetB specific IgY antibody levels were observed in eggs from rNetB(S254L) or Toxoid + rNetB(S254L) vaccinated hens compared to eggs from adjuvant control vaccinated hens (*p* < 0.001; Figure 2). Surprisingly, the level of anti-NetB specific IgY antibodies was also significant in eggs from hens vaccinated with toxoid alone (*p* < 0.01), despite the serum levels in the hens being similar to the adjuvant control hens. While the difference between serum and egg levels is interesting, it was not unexpected that the toxoid group had specific antibodies since the toxoid will have low levels of native NetB because it was made using a virulent strain that produces this toxin.

**Figure 3:**
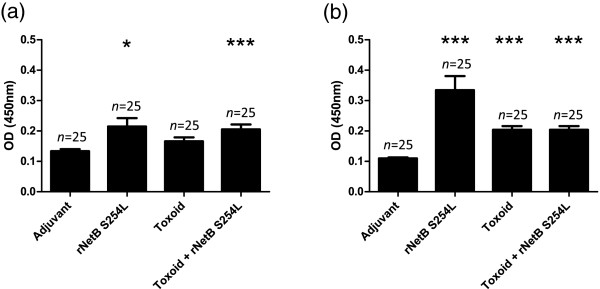
**Maternally derived anti-NetB specific IgY antibodies in progeny chickens. (a)** Anti-NetB IgY levels in 21 day-old chickens; **(b)** anti-NetB IgY levels in a separate group of 14 day-old chickens. Error bars represent SEM. *p < 0.05, ***p < 0.001.

The anti-NetB specific IgY levels in hatched chicks from vaccinated hens were assessed prior to challenge in the first trial (21 days of age; Figure 3a). The anti-NetB specific IgY antibody levels were significantly higher in those chicks from hens vaccinated with rNetB(S254L) alone (*p* < 0.05) or toxoid + rNetB(S254L) (*p* < 0.001) compared to the adjuvant control chicks. However, unlike the anti-NetB specific IgY antibody levels in eggs, the toxoid alone group of chicks did not have significantly higher levels than the adjuvant control birds (*p* > 0.1).

The anti-NetB specific IgY antibodies of chicks hatched from vaccinated hens for trial two were also assessed (14 days of age; Figure 3b). As before, the rNetB(S254L) and Toxoid + rNetB(S254L) groups had significantly higher levels of anti-NetB specific IgY antibodies than the adjuvant group (*p* < 0.001). However, unlike in the first trial, the toxoid group also had significantly higher levels of anti-NetB specific IgY antibodies compared the adjuvant control birds (*p* < 0.001). The difference in anti-NetB specific IgY antibody levels between the two trials could be attributed to the earlier time point of collection of the pre-challenge blood samples from the chicks (14 days compared to 21 days).

### Transferred antibodies partially protect birds from necrotic enteritis

**Figure 4:**
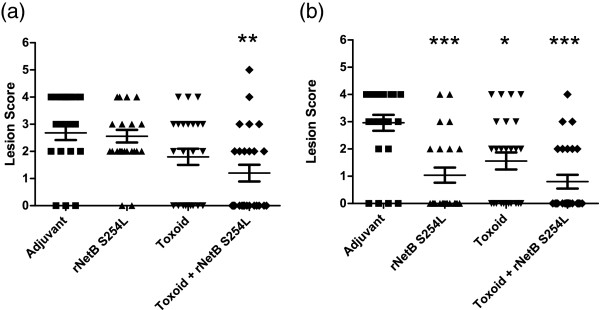
**Lesion scores in *C. perfringens *challenged progeny chickens.** The horizontal bars represent the average lesion score in each group. Each symbol represents an individual bird. **(a)** Trial 1: chickens challenged at day 21. **(b)** Trial 2 - chickens challenged at day 14. Error bars represent SEM. *n* = 25 per group. *p < 0.05, **p < 0.01, ***p < 0.001.

In trial one, in which birds were challenged at 21 days post-hatch, the toxoid + rNetB(S254L) vaccinated group had a 50% reduction in the number of birds with lesions (Table 1) and a significant reduction (54%) in average lesion score (*p* < 0.01) compared to the adjuvant control group (Figure 4a). However, neither the rNetB(S254L) nor the toxoid alone groups were protected compared to the adjuvant control group.

**Table 1:** Summary of trial results

**Trial**	**Group**	**Birds with NE**	**Average lesion score ± SEM**^**a**^	**Reduction in number of birds with NE (%)**	**Reduction in group average lesion score (%)**
**1**	Adjuvant	22/25	2.6 ± 0.3	-	
	NetB(S254L)	23/25	2.1 ± 0.2	-4.5	19
	Toxoid	16/25	1.8 ± 0.3	27	31
	Toxoid + NetB(S254L)	11/25	1.2 ± 0.3**	50	54
**2**	Adjuvant	21/25	3.0 ± 0.3	-	
	NetB(S254L)	10/25	1.0 ± 0.3***	52	67
	Toxoid	14/25	1.6 ± 0.3*	33	47
	Toxoid + NetB(S254L)	8/25	0.8 ± 0.3***	62	73

^a^Treatment groups were compared to adjuvant control group using Kruskal-Wallis one-way ANOVA with Dunns post test *denotes *p* < 0.05; **denotes *p* < 0.01; ***denotes *p* < 0.001.

In trial two birds were challenged at a younger age; at 14 days post-hatch. Similar to trial one, the toxoid + rNetB(S254L) group had a reduction in the number of birds with lesions (62%, Table 1) and a highly significant reduction (73%) in mean lesion score (*p* < 0.001) compared to the adjuvant control group (Figure 4b). However, unlike trial one, both the rNetB(S254L) and toxoid alone groups also had fewer birds with lesions (52% and 33% reduction, respectively) and a significant reduction in average lesion scores (*p* < 0.001 and *p* < 0.05, respectively) compared to the adjuvant control group.

## Discussion

This is the first study to demonstrate that passive transfer of antibodies from hens vaccinated with NetB-based vaccines can protect progeny from necrotic enteritis at the peak ages of disease susceptibility. It is anticipated that such vaccines would provide a valuable tool for the prevention of necrotic enteritis under field conditions. Previous studies that have investigated passive immunity against necrotic enteritis have been based on vaccines to induce alpha-toxin antibodies [5,14]. Although alpha-toxin vaccines can clearly provide some level of protection, both when used to directly vaccinate birds and when used for maternal vaccination, the antigen is not an essential virulence factor in the pathogenesis of necrotic enteritis [15]. We hypothesized that using the important necrotic enteritis virulence factor NetB, as either a single subunit vaccine or to enhance a conventional clostridial toxoid vaccine, was likely to provide higher levels of protection than alpha-toxin based vaccines.

The origins of the strains used to produce the maternal toxoid vaccines used in the study reported by Lovland et al. [14] are not reported and the *C. perfringens* strain used to generate the toxoid tested in the Crouch et al. study [5] was isolated from a cow. While this strain produces a large quantity of alpha-toxin [18] it most likely lacks other proteins involved in pathogenesis in chickens, including NetB. There is evidence that the choice of *C. perfringens* strain used for toxoid production is likely to influence the degree of protection that can be induced. The most direct evidence for this is the different degrees of efficacy noted by Lanckriet et al. [19] when comparing toxoids prepared from different strains. The importance of using chicken isolates of *C. perfringens* for vaccine preparation is supported by the finding of other potential virulence factors genetically linked to *netB*, a gene found almost exclusively in chicken isolates [20,21]. The strain used to generate the toxoid in this current study (EHE-NE18) is a strain that was isolated from a chicken with necrotic enteritis and is capable of causing disease under experimental conditions [15,22]. This strain not only produces alpha-toxin but also produces other proteins involved in disease, including NetB. It is therefore likely to contain a greater range of relevant antigens in toxoid preparations. Although EHE-NE18 does produce NetB, the level of the toxin produced under the in vitro culture conditions used in this current study was generally not sufficient to induce a strong anti-NetB immune response in vaccinated hens compared to the adjuvant control (Figure 1a). The addition of rNetB(S254L) to the toxoid preparation significantly increased the anti-NetB antibody levels in the hens and these antibody levels were also significantly higher in the chicks hatched from vaccinated hens (Figure 3). Transfer of specific antibodies is essential for the protection of young birds, since their immune system is not fully developed, especially the gut-associated lymphoid tissue, the maturation of which is not complete until two weeks of age [23]. The vaccination regime used in this study incorporated three vaccinations across six weeks with the final vaccination occurring when the hens were 26 weeks old. Although we saw no evidence of a drop of egg production in this study (data not shown) this vaccination regime is unlikely be the regime adopted by the poultry industry. Similar to other vaccines used in broiler breeders by the industry, the final vaccine most likely would be administered within the first 18 weeks post hatch to ensure that there is no impact on egg production.

Maternally derived antibodies in the progeny are at maximal levels at the time of hatch and gradually reduce in concentration as the birds mature. The key to the success of a maternal vaccination protocol is to ensure that there are sufficient antibodies circulating at the critical period when the birds are most susceptible to disease. The peak period when necrotic enteritis is a problem in broiler chickens is between 2 to 6 weeks post-hatch [24], hence we tested vaccine efficacy following challenge within this this critical period. Significant protection was seen in the progeny from hens vaccinated with toxoid plus rNetB(S254L) when challenged with a homologous strain (EHE-NE18) in-feed at day 14 and 21 (Figures 4a and b). Surprisingly, given previous results with directly vaccinated birds [12], significant protection was observed against in-feed challenge in the birds vaccinated with rNetB(S254L) alone (*p* < 0.001) and toxoid alone (*p* < 0.05) when challenged at 14 days (Figure 4b). This result may be due to higher levels of specific antibodies at the time of challenge, although the difference between the serum levels of rNetB(S254L) and toxoid at the time of challenge was not significant (*p* > 0.1). Even though significant protection was seen in progeny from hens vaccinated with rNetB(S254L) alone and toxoid alone at 14 days, only birds from toxoid plus rNetB(S254L) hens were significantly protected at both 14 and 21 days.

In conclusion, it has been shown that vaccination of broiler breeders with a *C. perfringens* toxoid preparation derived from a chicken necrotic enteritis strain and enhanced by the addition of non-toxic rNetB(S254L) protein, followed by subsequent transfer of maternal immunity to their progeny, provides significant protection against necrotic enteritis. The ease of delivery indicates that this type of vaccine may be a suitable candidate for use in the poultry industry in the near future.

## Competing interests

ALK, JIR and RJM have a patent on NetB and its use in vaccines: Clostridial Toxin NetB. US Patent no. 8,263,088 B2.

## Authors’ contributions

ALK, JIR, RJM conceived and designed the experiments. ALK, RWP, MEF, TLB, XY, RJM performed the experiments and analysed the data. ALK performed the statistical analysis. ALK drafted the paper and RJM and JIR modified and refined it. All authors read and approved the final manuscript.
